# Improving nurses’ alarm-management performance with a behavioral reminder

**DOI:** 10.1590/1980-220X-REEUSP-2025-0579en

**Published:** 2026-09-21

**Authors:** Serpil Topçu, Merve Kiymaç Sari, Melis Şen Yilmaz, Dilek Özdemir Ürkmez

**Affiliations:** 1 İstinye University, Faculty of Health Sciences, Department of Nursing, Istanbul, Türkiye.; 2 Fenerbahçe University, Faculty of Health Sciences, Department of Nursing, Istanbul, Türkiye.; 3 Biruni University, Faculty of Health Sciences, Department of Nursing, Istanbul, Türkiye.; 4 Prof. Dr. Cemil Taşçıoğlu City Hospital, General Intensive Care Unit, Istanbul, Türkiye.

**Keywords:** Clinical Alarms, Intensive Care Units, Patient Safety, Monitoring, Physiologic, Nursing Staff, Alarmes Clínicos, Unidades de Terapia Intensiva, Segurança do Paciente, Monitoramento Fisiológico, Recursos Humanos de Enfermagem

## Abstract

**Objective:**

To determine whether adding a behavioral reminder to the CEASE Bundle improves alarm-management performance and reduces alarm fatigue among intensive care nurses.

**Method:**

A controlled quasi-experimental pretest–posttest study was conducted with 115 nurses from adult intensive care units. Sixty-two nurses participated in the intervention group and 53 in the control group. Data were obtained with the Nurse Identification Form, CEASE Bundle, Alarm Type and Management Form, “Remind Me” Monitor Alarm Control Tool, and Alarm Fatigue Scale. CEASE provided the basis for observation and evaluation in both groups. Remind Me was used only by the intervention group for 15 days.

**Results:**

Compared with the control group, nurses in the intervention group demonstrated more favorable alarm-management behaviors and Alarm Fatigue Scale scores. Alarm response times decreased by approximately 15%–23% across alarm types, and the mean Alarm Fatigue Scale score in the intervention group increased from 68.67 ± 7.52 to 74.92 ± 4.22. Significant improvements were observed in several CEASE components, including communication, electrode and pulse oximeter care, adjustment of alarm limits, cancellation of unnecessary parameters, and identification of educational needs. In the multivariable analysis, exposure to the Remind Me intervention was independently associated with better alarm-management performance.

**Conclusion:**

Integrating the Remind Me behavioral reminder into routine monitoring practices was associated with more favorable alarm-management behaviors, shorter alarm response times, and reduced alarm fatigue among intensive care nurses. These findings suggest that low-cost reminder-based strategies may strengthen evidence-based alarm-management practices.

**Clinical trial registration:**

NCT06403397 (URL: https://clinicaltrials.gov/study/NCT06403397).

## RESUMO

**Objetivo:**

Determinar se a adição de um lembrete comportamental ao Bundle CEASE melhora o desempenho no gerenciamento de alarmes e reduz a fadiga de alarmes entre enfermeiros de unidades de terapia intensiva.

**Método:**

Estudo quase experimental controlado, com desenho pré-teste–pós-teste, realizado com 115 enfermeiros de unidades de terapia intensiva para adultos. Sessenta e dois enfermeiros compuseram o grupo intervenção e 53, o grupo controle. Os dados foram obtidos por meio do Formulário de Identificação do Enfermeiro, do Bundle CEASE, do Formulário de Tipo e Gerenciamento de Alarmes, da Ferramenta de Controle de Alarmes de Monitor “Remind Me” e da Escala de Fadiga de Alarmes. O CEASE fundamentou a observação e a avaliação em ambos os grupos. O Remind Me foi utilizado apenas pelo grupo intervenção, durante 15 dias.

**Resultados:**

Em comparação com o grupo controle, os enfermeiros do grupo intervenção apresentaram comportamentos mais favoráveis de gerenciamento de alarmes e melhores escores na Escala de Fadiga de Alarmes. Os tempos de resposta aos alarmes diminuíram aproximadamente entre 15% e 23% conforme o tipo de alarme, e o escore médio da Escala de Fadiga de Alarmes no grupo intervenção aumentou de 68,67 ± 7,52 para 74,92 ± 4,22. Foram observadas melhorias significativas em vários componentes do CEASE, incluindo comunicação, cuidados com eletrodos e oxímetro de pulso, ajuste dos limites de alarme, cancelamento de parâmetros desnecessários e identificação de necessidades educacionais. Na análise multivariável, a exposição à intervenção Remind Me associou-se independentemente a melhor desempenho no gerenciamento de alarmes.

**Conclusão:**

A integração do lembrete comportamental Remind Me às práticas rotineiras de monitoramento associou-se a comportamentos mais favoráveis de gerenciamento de alarmes, menores tempos de resposta e redução da fadiga de alarmes entre enfermeiros de unidades de terapia intensiva. Esses achados indicam que estratégias de baixo custo baseadas em lembretes podem fortalecer práticas de gerenciamento de alarmes fundamentadas em evidências.

## INTRODUCTION

Bedside patient monitors are widely used to continuously monitor vital signs in critically ill patients. These devices track basic parameters such as electrocardiography (ECG), blood pressure, pulse oximetry, and respiration, as well as advanced hemodynamic variables, including central venous and pulmonary artery pressures^(1,2,3)^. When abnormal values are detected, the monitors emit visual and audible alerts intended to attract clinicians’ attention^(4,5)^.

According to The Joint Commission’s (TJC) 2013 Sentinel Event Alert, an intensive care unit may generate thousands of alarms per day, 85%–99% of which are clinically insignificant^(6,7)^. This excessive alarm burden can lead clinicians to silence or ignore alarms, thereby posing a serious threat to patient safety^(3,8,9)^. The TJC report linked inadequate alarm management to patient deaths and subsequently included clinical alarm safety among its 2014 National Patient Safety Goals^(5,7,10)^.

Alarm-related errors may arise when alarms are missed, staff become desensitized (alarm fatigue), knowledge is insufficient, responses are delayed, interfaces are difficult to use, equipment malfunctions, or staffing is inadequate^(4,5,7,11,12,13)^. Intensive care nurses work with several devices at the same time, each with different limits and priority levels; consequently, repeated alarm sounds can gradually lose their salience and slow the response to clinically important signals^(3,8,14)^. Safety guidance, therefore, emphasizes individualized alarm settings together with procedures tailored to each unit^(1,5,6)^. The CEASE Bundle—Communication, Electrodes, Appropriateness, Setup, and Education—provides a structured approach to patient-specific alarm management and was introduced by Lewis and Oster^(15)^ in 2019. Earlier applications have been linked to fewer alarms and lower alarm fatigue^(9,15)^, although the evidence remains limited. Against this background, we examined whether adding the “Remind Me” behavioral cue to CEASE could improve nurses’ alarm-management performance and reduce alarm fatigue.

## METHOD

### Study Design

We used a controlled, quasi-experimental pretest–posttest design to examine changes in alarm-management behavior, alarm fatigue, and response indicators after the CEASE Bundle was paired with a behavioral reminder. Reporting followed the Transparent Reporting of Evaluations with Nonrandomized Designs (TREND) statement.

### Study Hypotheses

Two hypotheses were specified. First, nurses exposed to Remind Me alongside the CEASE Bundle would demonstrate better alarm-management behaviors and response outcomes than those receiving usual practice. Second, the intervention would be associated with lower alarm fatigue.

### Population

The target population comprised nurses employed in the hospital’s adult intensive care units.

### Setting

Data collection took place from May to September 2024 in the adult intensive care units of a tertiary training and research hospital in Istanbul, Türkiye.

### Selection Criteria

Participation required nurses to be actively working in the unit during data collection, volunteer for the study, be available rather than on leave, and provide written informed consent. Nurses allocated to the intervention group also had to agree to use Remind Me. Nurses were excluded if they were on annual, sick, or administrative leave; declined participation; failed to complete either the pretest or posttest; or were assigned to the unit only temporarily.

### Sample

Of the 142 nurses employed in the participating intensive care units, 115 entered the study: 62 in the intervention group and 53 in the control group. Allocation was made by the intensive care unit rather than by an individual nurse to limit contamination between study conditions. The Anesthesiology and Reanimation I Unit formed the intervention cluster (n = 62). The control group comprised the Anesthesiology and Reanimation II Unit (n = 16), Cardiovascular Surgery Unit (n = 18), and Coronary Intensive Care Unit (n = 19).

### Study Variables

The study variables included demographic and professional characteristics (age, gender, educational level, years of professional experience, weekly working hours, prior alarm training, and unit assignment) and behavioral indicators related to alarm management. Behavioral variables included alarm suspension practices, alarm-limit adjustment, electrode and sensor management, escalation and communication processes, and other observable alarm-management actions. Operational variables included alarm frequency, repetition patterns, duration, and response times for white, yellow, and red alarms. In the monitoring system used in this study, white alarms represented low-priority technical or informational alerts, yellow alarms represented medium-priority physiological warnings requiring clinical assessment, and red alarms represented high-priority critical alarms requiring an immediate clinical response. Perceptual variables comprised nurses’ levels of alarm fatigue. Together, these variables represented the demographic, behavioral, perceptual, and operational components relevant to evaluating alarm-management performance and alarm fatigue.

### Data Collection Instruments

Nurse Identification Form: The researchers developed this 14-item form based on the literature to collect information on nurses’ demographic and professional characteristics^(1,14,15)^.

CEASE Bundle: Developed by Lewis and Oster^(15)^, the CEASE Bundle is a nurse-driven, evidence-based, patient-specific monitoring bundle designed to reduce alarm fatigue. In the bundle, Communication (C) refers to communication about alarm parameters and alarm-related problems; Electrodes (E) refers to daily electrode replacement; Appropriateness (A) refers to evaluating the appropriateness of monitoring; Setup (S) refers to patient-specific alarm-parameter settings; and Education (E) refers to ongoing education. In this study, the CEASE Bundle guided the observation and evaluation of nurses’ alarm-management behaviors in both the intervention and control groups.

Alarm Type and Management Form: The researchers developed this form based on a review of the literature^(5,16)^. It included sections on alarm type, definition, parameters, alarm response, number of repetitions, and response time.

Monitor Alarm Control Tool—“Remind Me”: The researchers developed this tool using recommendations from the Association for the Advancement of Medical Instrumentation (AAMI), The Joint Commission (TJC), and the American Association of Critical-Care Nurses (AACN)^(6,9,16)^. It contains four sections covering monitor checks, the accuracy of alarm settings, recognition of alarm types, and documentation. Within CEASE, the tool was intended to serve as a practical prompt for the Education component and to keep key alarm-management steps visible during routine care.

Alarm Fatigue Scale: Ashrafi et al.^(17)^ developed the 23-item Alarm Fatigue Assessment Scale, with responses recorded on a five-point Likert scale. The Turkish adaptation contains 21 items and yields scores from 0 to 84; lower scores reflect more severe alarm fatigue^(14)^.

### Data Collection

Baseline information was obtained through face-to-face interviews and direct observation. All participating nurses completed the Nurse Identification Form and the Alarm Fatigue Scale. In each unit, two researchers then observed bedside monitors continuously for 24 hours through the central monitoring system and used the CEASE Bundle together with the Alarm Type and Management Form to conduct a root-cause assessment. Observations covered communication, electrode care, appropriateness of monitoring, alarm setup, and education needs. To limit observation-related bias, nurses were informed that findings would be recorded anonymously at the unit level and would not be used to evaluate individual performance. CEASE was therefore used in both groups as the common assessment framework, whereas Remind Me was introduced only in the intervention units. After the 15-day study period, the Alarm Fatigue Scale was administered again in both groups.

### Implementation in the Intervention Group

Following the baseline observation, the research team introduced Remind Me to nurses in the intervention unit. Short orientation sessions explained how the tool should be used and why each item was relevant. A PVC-laminated copy was placed beneath each monitor so that it remained visible during routine care. Nurses used the tool for 15 days. During this period, charge nurses provided occasional reminders, while the researchers otherwise did not influence clinical practice. At the end of the intervention, a second 24-hour observation was completed with the CEASE Bundle and the Alarm Type and Management Form.

### Implementation in the Control Group

Nurses in the control units continued their usual monitor-alarm practices. After the same 15-day interval, these units underwent a second 24-hour observation using the CEASE Bundle and the Alarm Type and Management Form.

### Data Analysis

Analyses were performed in IBM SPSS Statistics 22.0 (IBM Corp., Armonk, NY, USA). Continuous data were summarized with means and standard deviations, and categorical data with frequencies and percentages. Distributional assumptions were examined using the Shapiro–Wilk test. Depending on normality, within-group comparisons used either the paired-samples t test or the Wilcoxon signed-rank test, whereas between-group comparisons used the independent-samples t test or the Mann–Whitney U test. Categorical outcomes were assessed with chi-square or McNemar tests. A multivariable logistic regression model was fitted to identify factors related to improvement in alarm management. Statistical significance was defined as p < 0.05.

### Ethical Aspects

The study was conducted in accordance with national and international ethical guidelines and was approved by the Research Ethics Committee of Fenerbahçe University (April 16, 2024; Protocol No. 56.2024fbu). Written informed consent was obtained from all participants. The study complied with the principles of the Declaration of Helsinki. After data collection was completed, the Remind Me tool was made available to the control units.

### Use of Artificial Intelligence

Artificial intelligence tools were used only for language editing and formatting. The authors take full responsibility for the content and conclusions. No artificial intelligence tools were used for data analysis or interpretation.

## RESULTS

**Figure 1:**
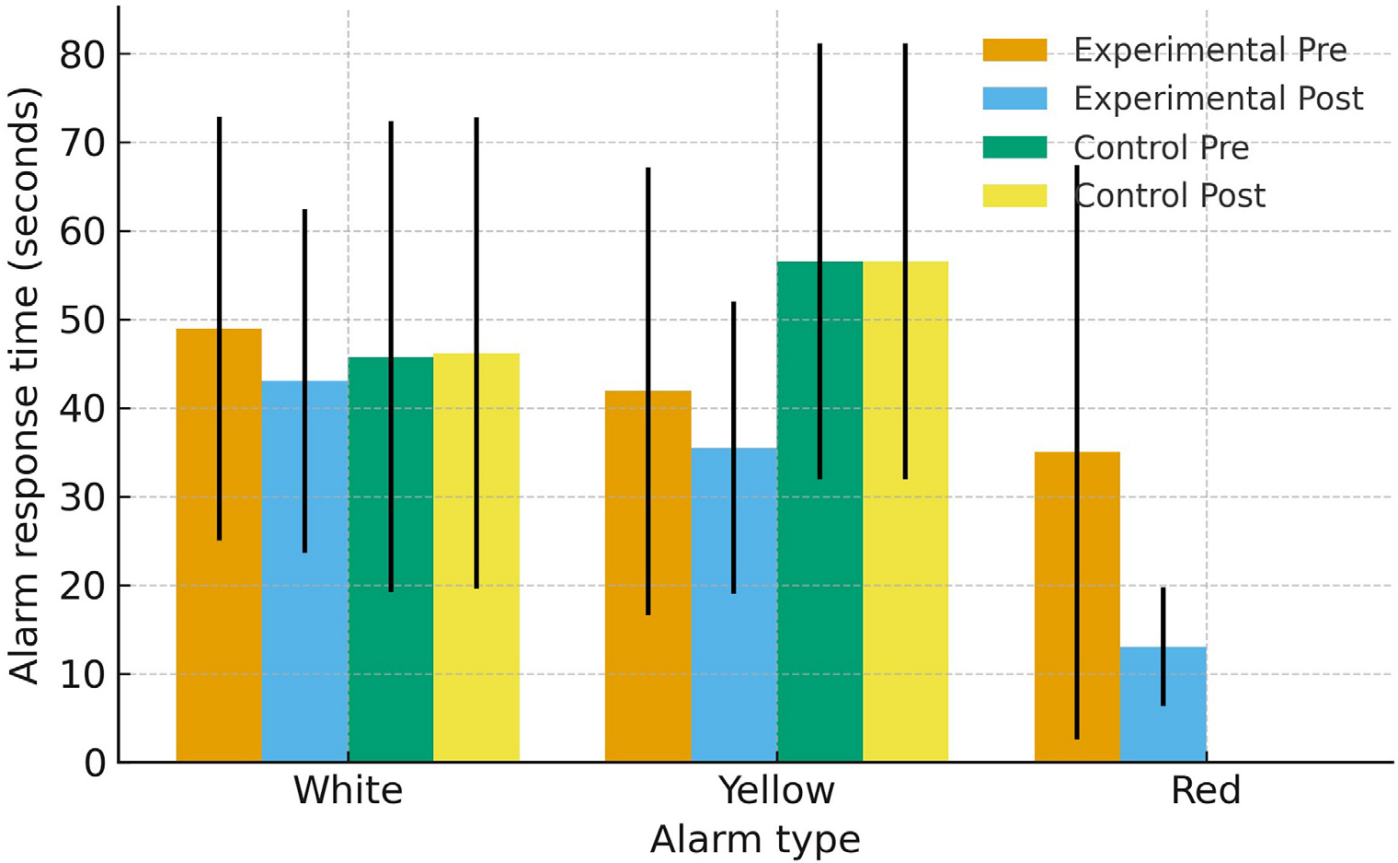
Alarm response times (seconds) for white, yellow, and red alarms among intensive care nurses – Istanbul, Türkiye, 2024.

No significant differences were found between the intervention and control groups in age (25.07 ± 2.44 vs. 26.67 ± 4.24 years), gender, marital status, parental status, educational level, total clinical experience, clinical role, or perceptions of monitor alarms (p > 0.05). Postintervention CEASE indicators showed that nurses in the intervention group suspended alarms before procedures, canceled unnecessary parameters, and adjusted alarm limits more frequently than those in the control group (p < 0.05; Table 1A). Pre–post comparisons in the intervention group showed significant improvements in the correct identification of white and yellow alarms, patient-specific parameter adjustment, the number of repeated alarms, and response times (p < 0.05; Table 1B); reductions in response times are illustrated in Figure 1. No statistically significant pre–post changes were observed in the control group (p > 0.05). Because no red-alarm events occurred in the control group during the observation period, red-alarm response-time outcomes are presented descriptively for the intervention group only and recorded as not applicable (N/A) for the control group in Table 1B.

**Table 1:** Postintervention CEASE behavioral indicators, pre–post alarm-response outcomes, and predictors of improved alarm-management performance among intensive care nurses (N = 115) – Istanbul, Türkiye, 2024.

A. Postintervention between-group comparison of CEASE* behavioral indicators			
**CEASE component**	**Intervention group, postintervention, % (n = 62)**	**Control group, postintervention, % (n = 53)**	**p^†^**
Alarm suspended before procedures	83.3	61.2	**0.041**
Unnecessary parameters canceled	68.4	50.0	**0.046**
Alarm limits adjusted as needed	76.3	58.9	**0.039**
**B. Pre–post alarm-response performance by alarm type**			
**Alarm type and response indicator**	**Intervention group, pre → post**	**Control group, pre → post**	**p^‡^**
White – Correct identification	82.0% → 88.5%	69.2% → 69.2%	**0.040**
White – Patient-specific parameters adjusted	78.7% → 96.7%	65.4% → 65.4%	**0.001**
White – Alarm resolved	13.1% → 37.7%	(No data)	N/A^§^
White – Repeated alarms (mean ± SD)	18.38 ± 5.74 → 17.00 ± 5.27	19.73 ± 5.90 → 19.73 ± 5.90	**0.009**
White – Response time, s (mean ± SD)	43.0 ± 19.4 → 35.2 ± 15.1	45.7 ± 26.6 → 46.1 ± 26.6	**0.018**
Yellow – Correct identification	49.2% → 62.3%	67.3% → 68.7%	**0.002**
Yellow – Patient-specific parameters adjusted	86.9% → 96.7%	76.9% → 76.9%	**0.004**
Yellow – Alarm resolved	No data → 29.5%	(No data)	N/A^§^
Yellow – Repeated alarms (mean ± SD)	15.31 ± 3.76 → 13.80 ± 3.24	18.19 ± 5.74 → 18.19 ± 5.74	**0.001**
Yellow – Response time, s (mean ± SD)	35.5 ± 16.5 → 30.1 ± 13.8	56.5 ± 24.6 → 56.5 ± 24.6	**0.021**
Red – Correct identification	16.4% → 18.0%	(No red alarms)	N/A^§^
Red – Response time, s (mean ± SD)	13.0 ± 6.7 → 10.0 ± 4.3	(No data)	N/A^§^
**C. Multivariable predictors of improved alarm-management performance**			
**Independent variable**	**^||^β (SE)**	**^¶^OR (95% CI)**	p**
Remind Me intervention	1.37 (0.52)	3.95 (1.38–11.32)	**0.008**
Experience (years)	0.06 (0.03)	1.06 (1.00–1.13)	**0.048**
Prior alarm training (yes)	0.85 (0.41)	2.34 (1.05–5.24)	**0.039**
Weekly working hours	–0.04 (0.02)	0.96 (0.92–0.99)	**0.042**

*CEASE = Communicate, Electrodes, Appropriateness, Setup, Education;

^†^McNemar and chi-square tests;

^‡^Wilcoxon signed-rank and Mann–Whitney U tests;

^§^not applicable (N/A);

^||^β = beta coefficient; SE = standard error;

^¶^OR = odds ratio; CI = confidence interval;

**binary logistic regression model.

Statistical significance was set at p < 0.05.

Multivariable logistic regression analysis showed that the Remind Me intervention was associated with improved alarm-management performance (OR = 3.95, 95% CI: 1.38–11.32, p = 0.008). Professional experience (OR = 1.06, 95% CI: 1.00–1.13, p = 0.048) and prior alarm training (OR = 2.34, 95% CI: 1.05–5.24, p = 0.039) were also positively associated with improved performance, whereas weekly working hours were inversely associated with improvement (OR = 0.96, 95% CI: 0.92–0.99, p = 0.042) (Table 1C).

**Figure 2:**
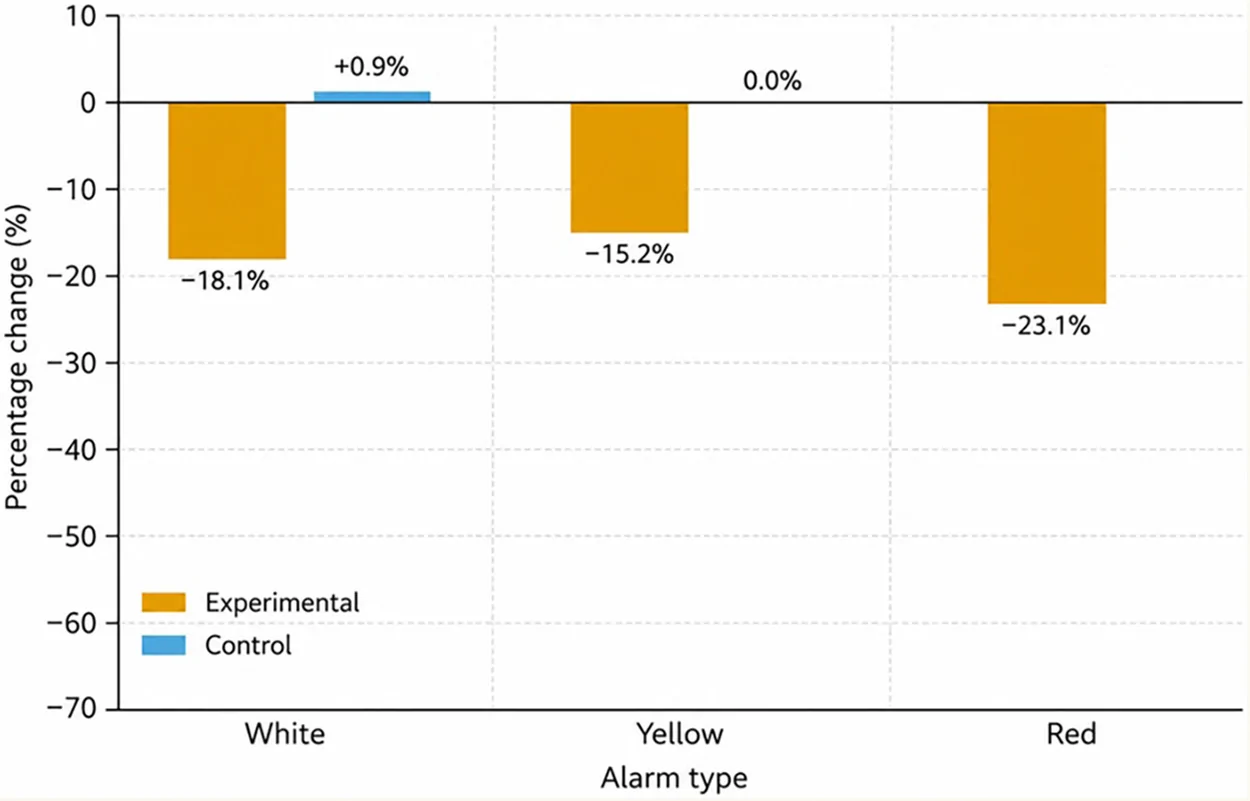
Percentage change in alarm response times from pre- to postintervention by alarm type among intensive care nurses – Istanbul, Türkiye, 2024.

Figure 2 shows that response times decreased for white, yellow, and red alarms in the intervention group, whereas the control group showed minimal or no change for white and yellow alarms. The percentage change in red-alarm response time was not calculated for the control group because no red alarms were observed during the study period.

Alarm Fatigue Scale scores increased in the intervention group from pretest (68.67 ± 7.52) to posttest (74.92 ± 4.22), whereas no statistically significant change was observed in the control group (Table 2). The posttest scores differed significantly between the groups (p = 0.005). Significant between-group differences were also observed in the following CEASE components: alarm suspension before procedures, notification of the primary nurse, electrode and pulse oximeter care, cancellation of unnecessary parameters, appropriate adjustment of alarm limits, adjustment of settings according to the patient’s condition, and identification of the need for re-education (p < 0.05; Table 2).

**Table 2:** Comparison of Alarm Fatigue Scale scores and significant CEASE components among intensive care nurses (N = 115) – Istanbul, Türkiye, 2024.

Parameter	Intervention (%/mean ± SD)	Control (%/mean ± SD)	p*
**Alarm Fatigue Scale**			
Pretest score	68.67 ± 7.52	70.92 ± 8.69	**< 0.001**
Posttest score	74.92 ± 4.22	71.08 ± 8.72	**0.005**
**Significant CEASE^†^ components p^‡^**			
Alarm suspended before procedures	93.4% / 74.9 ± 4.2	78.8% / 71.0 ± 6.1	**0.041**
Alarm reported to the primary nurse	85.2% / 75.2 ± 3.9	63.5% / 70.5 ± 6.0	**< 0.001**
Electrode and pulse oximeter care	85.2% / 74.8 ± 4.0	80.8% / 70.9 ± 6.1	**0.049**
Unnecessary parameters canceled	83.6% / 75.0 ± 3.8	76.9% / 70.1 ± 6.0	**0.046**
Alarm limits adjusted	85.2% / 74.7 ± 4.3	78.8% / 71.5 ± 5.9	**0.025**
Settings adjusted according to condition	83.6% / 74.5 ± 4.1	76.9% / 70.9 ± 6.3	**0.039**
Need for re-education identified	78.7% / 75.1 ± 3.9	63.5% / 69.8 ± 6.5	**0.031**

*Paired-samples t test;

†CEASE = Communicate, Electrodes, Appropriateness, Setup, Education;

^‡^McNemar and chi-square tests.

Statistical significance was set at p < 0.05.

## DISCUSSION

Intensive care units generate large numbers of non-actionable alarms each day, contributing to both alarm fatigue among nurses and delayed responses to true clinical emergencies^(6,10,11,18)^. The Joint Commission’s Sentinel Event Alert 50 recommends conducting a situational analysis and developing unit-specific protocols to improve alarm safety^(5)^. Consistent with these recommendations, integrating Remind Me—a low-cost behavioral reminder designed to reinforce the educational component of the CEASE Bundle—into routine monitoring practices was associated with shorter alarm response times, improved alarm-management behaviors, and more favorable Alarm Fatigue Scale scores.

In the intervention group, alarm response times decreased by approximately 15%–23% across alarm types. Lewis and Oster^(15)^ reported a 31% reduction in total alarm frequency following implementation of the CEASE Bundle^(15)^. Although the present study focused on alarm response times, whereas Lewis and Oster^(15)^ examined alarm frequency, the findings reflect improvements in different but complementary dimensions of alarm management. The CEASE Bundle was used as the observation and evaluation framework in both groups; however, response times improved only in the intervention group, in which the Remind Me tool was implemented. This finding suggests that the reminder may further strengthen routine alarm-management practices.

The improvement in alarm-management behaviors suggests that Remind Me may have helped nurses perform routine alarm-management practices more consistently. The effect was particularly evident in directly observable behaviors, such as suspending alarms before procedures, canceling unnecessary parameters, and adjusting alarm limits in response to changes in patients’ clinical conditions. These behaviors require not only technical knowledge of monitor settings but also sustained attention, timely reassessment, and clinical decision-making. Previous studies have shown that nurses’ responses to monitor alarms are associated with alarm relevance, the burden of false alarms, and alarm-management practices^(18)^. Evidence-based alarm-management strategies may reduce alarm burden^(19,20)^, whereas alarm-management training may reduce alarm fatigue and improve nurses’ awareness and behaviors^(21)^. Contextual factors, including workload, alarm burden, and monitor customization, may also influence alarm-management processes^(1,10,13)^, and workload has been shown to impair alarm perception and localization performance^(22)^.

When evaluated by alarm type, the effect of the Remind Me intervention was particularly evident for low- and medium-priority alarms. For white alarms, the increase in patient-specific parameter adjustment indicates that these alarms were not only recognized but also reassessed in relation to the patient’s clinical condition. For yellow alarms, concurrent improvements in correct identification, appropriate parameter adjustment, the number of repeated alarms, and response time suggest that Remind Me may have promoted a more systematic response to medium-priority physiological deviations. These findings are consistent with studies reporting that alarm-management training and structured alarm-management strategies may reduce alarm burden and improve alarm-management behaviors^(19,21)^. For red alarms, the decrease in response time from 13.0 to 10.0 seconds is clinically noteworthy because it suggests a faster response to high-priority alarms. However, because comparable red-alarm observations were unavailable for the control group, this finding should be interpreted as a clinical trend rather than a statistical comparison. Overall, this pattern suggests that Remind Me may improve the management of low- and medium-priority alarms, reduce exposure to repeated alarms, and preserve clinical attention for high-priority alarms.

The multivariable analysis further supports the independent association between the Remind Me intervention and improved alarm-management performance. Membership in the intervention group was associated with nearly fourfold higher odds of improved alarm-management performance, while professional experience and prior alarm training were positive predictors. In contrast, longer weekly working hours were associated with lower odds of improved performance, indicating that alarm management is shaped not only by individual knowledge and awareness but also by working conditions and workload. This finding is consistent with studies showing that alarm-management training may reduce alarm fatigue and improve awareness and behavior^(21)^, as well as research indicating that alarm burden, monitor customization, and other contextual factors may influence alarm-management processes^(1,13,22)^.

Alarm fatigue reduces nurses’ sensitivity to true emergency signals, thereby increasing the risk of missed critical alarms and threatening patient safety^(23,24)^. Nonactionable and repeated alarms are associated with distraction, desensitization, and delayed responses^(1,4,6,13)^, and nurses have reported that alarm fatigue delays their responses to alarms^(5,10)^. Because a higher Alarm Fatigue Scale score represents a more favorable outcome, the higher posttest score in the intervention group suggests that Remind Me may have reduced alarm fatigue. Adherence to several CEASE-related behaviors exceeded 75% for multiple indicators in the control group, suggesting that the CEASE Bundle may have supported basic alarm-management behaviors. However, the higher adherence rates in the intervention group suggest that Remind Me may have further facilitated the translation of CEASE recommendations into routine care. Thus, the favorable change in Alarm Fatigue Scale scores occurred alongside observable improvements in CEASE-related behaviors.

Taken together, these findings suggest that Remind Me may have functioned as a simple bedside cue that facilitated the translation of CEASE recommendations into routine practice. Behavioral reminders may help sustain the effects of education, particularly when the impact of structured training diminishes over time^(20,21)^. Visual cues and reminder-based interventions have also been reported to strengthen evidence-based alarm-management behaviors by supporting nurses’ situational awareness and alarm-prioritization skills^(11)^. Therefore, low-cost behavioral reminders such as Remind Me may be a practical strategy for supporting sustainable alarm management under real-world intensive care conditions.

## LIMITATIONS

Because allocation followed existing unit structures rather than randomization, residual confounding cannot be ruled out. We sought to reduce this risk by choosing units with comparable patient populations and monitoring systems.

Awareness of being observed may also have altered nurses’ behavior despite the assurance that data would be analyzed anonymously at the unit level. In addition, we did not record how often or how consistently nurses consulted Remind Me. Although the tool was kept visible and its use was reinforced during brief meetings, future research should include an objective measure of adherence.

Red alarms were uncommon during observation. Their response times were therefore summarized descriptively and were not included in inferential analyses.

## CONCLUSION

Pairing Remind Me with the CEASE Bundle appeared to support its use in routine intensive care practice. Nurses in the intervention unit more often reported alarms to the primary nurse, adjusted alarm settings to the individual patient, removed unnecessary parameters, and recognized when further education was needed. They also responded more quickly to alarms and had more favorable postintervention Alarm Fatigue Scale scores.

Both prespecified hypotheses were supported. The findings indicate that the combined approach can improve alarm-management processes and may lessen alarm fatigue. Although total alarm counts were not the primary outcome, the observed changes in behavior, repeated-alarm indicators, and response patterns point to more focused alarm handling.

A visible bedside prompt is inexpensive and easy to incorporate into existing workflows. Used alongside structured education, such reminders may help nurses retain key alarm-management steps, prioritize alarms more effectively, and reduce the cumulative burden of alarms in intensive care.

## Data Availability

The datasets generated and/or analyzed during the current study are available from the corresponding author upon reasonable request.
