# Supplementary material for: Improving nurses’ alarm-management performance with a behavioral reminder
Source: Rev Esc Enferm USP. 2026 Sep 21;60:e20250579. doi: 10.1590/1980-220X-REEUSP-2025-0579en (PMC13600734; doi:10.1590/1980-220X-REEUSP-2025-0579en)
Supplement: Translation

# Translation: Mejora del desempeño del personal de enfermería en la gestión de alarmas mediante un recordatorio conductual

**DOI:** 10.1590/1980-220X-REEUSP-2025-0579es

**Authors:** Serpil Topçu, Merve Kiymaç Sari, Melis Şen Yilmaz, Dilek Özdemir Ürkmez

**Affiliations:** 1 İstinye University, Faculty of Health Sciences, Department of Nursing, Istanbul, Türkiye.; 2 Fenerbahçe University, Faculty of Health Sciences, Department of Nursing, Istanbul, Türkiye.; 3 Biruni University, Faculty of Health Sciences, Department of Nursing, Istanbul, Türkiye.; 4 Prof. Dr. Cemil Taşçıoğlu City Hospital, General Intensive Care Unit, Istanbul, Türkiye.

**Keywords:** Alarmas Clínicas, Unidades de Cuidados Intensivos, Seguridad del Paciente, Monitoreo Fisiológico, Personal de Enfermería

## Abstract

**Objetivo:**

Determinar si la incorporación de un recordatorio conductual al Bundle CEASE mejora el desempeño en la gestión de alarmas y reduce la fatiga por alarmas entre el personal de enfermería de unidades de cuidados intensivos.

**Método:**

Estudio cuasiexperimental controlado, con diseño pretest–postest, realizado con 115 profesionales de enfermería de unidades de cuidados intensivos de adultos. Sesenta y dos participaron en el grupo de intervención y 53 en el grupo control. Los datos se obtuvieron mediante el Formulario de Identificación del Personal de Enfermería, el Bundle CEASE, el Formulario de Tipo y Gestión de Alarmas, la Herramienta de Control de Alarmas del Monitor “Remind Me” y la Escala de Fatiga por Alarmas. CEASE sirvió de base para la observación y evaluación en ambos grupos. Remind Me se utilizó únicamente en el grupo de intervención durante 15 días.

**Resultados:**

En comparación con el grupo control, el personal de enfermería del grupo de intervención mostró conductas más favorables de gestión de alarmas y mejores puntuaciones en la Escala de Fatiga por Alarmas. Los tiempos de respuesta disminuyeron aproximadamente entre un 15% y un 23%, según el tipo de alarma, y la puntuación media de la Escala de Fatiga por Alarmas en el grupo de intervención aumentó de 68,67 ± 7,52 a 74,92 ± 4,22. Se observaron mejoras significativas en varios componentes de CEASE, entre ellos la comunicación, el cuidado de electrodos y del pulsioxímetro, el ajuste de los límites de alarma, la cancelación de parámetros innecesarios y la identificación de necesidades educativas. En el análisis multivariable, la exposición a la intervención Remind Me se asoció de forma independiente con un mejor desempeño en la gestión de alarmas.

**Conclusión:**

La integración del recordatorio conductual Remind Me en las prácticas habituales de monitorización se asoció con conductas más favorables de gestión de alarmas, menores tiempos de respuesta y una reducción de la fatiga por alarmas entre el personal de enfermería de unidades de cuidados intensivos. Estos hallazgos indican que las estrategias de bajo costo basadas en recordatorios pueden fortalecer las prácticas de gestión de alarmas fundamentadas en la evidencia.

**Registro del ensayo clínico:**

NCT06403397 (URL: https://clinicaltrials.gov/study/NCT06403397).

## INTRODUCCIÓN

Los monitores de cabecera se utilizan ampliamente para monitorear de forma continua los signos vitales de los pacientes en estado crítico. Estos dispositivos registran parámetros básicos, como la electrocardiografía (ECG), la presión arterial, la oximetría de pulso y la respiración, así como variables hemodinámicas avanzadas, entre ellas la presión venosa central y la presión arterial pulmonar^(1,2,3)^. Cuando detectan valores anormales, emiten alertas visuales y sonoras destinadas a captar la atención del personal clínico^(4,5)^. Según la Alerta de Evento Centinela de 2013 de The Joint Commission (TJC), una unidad de cuidados intensivos puede generar miles de alarmas al día, de las cuales entre el 85% y el 99% carecen de relevancia clínica^(6,7)^. Esta carga excesiva puede llevar a los profesionales de la salud a silenciar o ignorar las alarmas, lo que supone un riesgo grave para la seguridad del paciente^(3,8,9)^. TJC relacionó la gestión inadecuada de las alarmas con muertes de pacientes y posteriormente incluyó la seguridad de las alarmas clínicas entre los Objetivos Nacionales de Seguridad del Paciente de 2014^(5,7,10)^.

Los errores relacionados con las alarmas pueden producirse cuando estas no se detectan, el personal se desensibiliza a ellas (fatiga por alarmas), el conocimiento es insuficiente, la respuesta se retrasa, las interfaces resultan difíciles de utilizar, los equipos fallan o la dotación de personal es inadecuada^(4,5,7,11,12,13)^. El personal de enfermería de cuidados intensivos trabaja simultáneamente con varios dispositivos, cada uno con límites y niveles de prioridad diferentes; por ello, la repetición de los sonidos de alarma puede reducir progresivamente su capacidad para captar la atención y retrasar la respuesta a señales clínicamente importantes^(3,8,14)^. Las recomendaciones de seguridad hacen hincapié en individualizar los ajustes de las alarmas y desarrollar procedimientos específicos para cada unidad^(1,5,6)^. El paquete CEASE —Comunicación, Electrodos, Adecuación, Configuración y Educación (Communication, Electrodes, Appropriateness, Setup, and Education)— ofrece un enfoque estructurado para la gestión de alarmas adaptada a cada paciente y fue presentado por Lewis y Oster^(15)^. Su implementación se ha asociado con una reducción de la frecuencia de las alarmas y de la fatiga por alarmas^(9,15)^, aunque la evidencia aún es limitada. En este contexto, evaluamos si añadir el recordatorio conductual «Remind Me» al paquete CEASE podía mejorar el desempeño del personal de enfermería en la gestión de alarmas y reducir la fatiga por alarmas.

## MÉTODO

### Diseño del Estudio

Se empleó un diseño cuasiexperimental controlado con mediciones pretest y postest para evaluar los cambios en las conductas de gestión de alarmas, la fatiga por alarmas y los indicadores de respuesta tras combinar el paquete CEASE con un recordatorio conductual. El estudio se informó de acuerdo con la declaración TREND (Transparent Reporting of Evaluations with Nonrandomized Designs).

### Hipótesis del Estudio

Se formularon dos hipótesis. En primer lugar, el personal de enfermería expuesto a «Remind Me» junto con el paquete CEASE presentaría mejores conductas de gestión de alarmas y mejores resultados de respuesta que el personal que continuara con la práctica habitual. En segundo lugar, la intervención se asociaría con una menor fatiga por alarmas.

### Población

La población objetivo estuvo constituida por el personal de enfermería que trabajaba en las unidades de cuidados intensivos de adultos del hospital.

### Entorno

La recolección de datos se llevó a cabo entre mayo y septiembre de 2024 en las unidades de cuidados intensivos de adultos de un hospital de formación e investigación de tercer nivel en Estambul, Turquía.

### Criterios de Selección

Podían participar los profesionales de enfermería que trabajaran activamente en la unidad durante la recolección de datos, aceptaran participar voluntariamente, no se encontraran de licencia y otorgaran su consentimiento informado por escrito. Quienes fueron asignados al grupo de intervención también debían aceptar el uso de «Remind Me». Se excluyó al personal que se encontraba de licencia anual, médica o administrativa; rechazó participar; no completó las evaluaciones pretest o postest; o estaba asignado temporalmente a la unidad durante el periodo del estudio.

### Muestra

De los 142 profesionales de enfermería que trabajaban en las unidades de cuidados intensivos participantes, se incluyeron 115: 62 en el grupo de intervención y 53 en el grupo control. La asignación se realizó por conglomerados de unidad, y no de forma individual, para preservar la integridad de los datos y evitar la contaminación entre grupos. La Unidad de Anestesiología y Reanimación I constituyó el grupo de intervención (n = 62). El grupo control estuvo integrado por la Unidad de Anestesiología y Reanimación II (n = 16), la Unidad de Cirugía Cardiovascular (n = 18) y la Unidad de Cuidados Intensivos Coronarios (n = 19).

### Variables del Estudio

Las variables del estudio incluyeron características demográficas y profesionales (edad, género, nivel educativo, años de experiencia profesional, horas de trabajo semanales, capacitación previa sobre alarmas y unidad de adscripción), así como indicadores conductuales relacionados con la gestión de alarmas. Las variables conductuales comprendieron las prácticas de suspensión de alarmas, el ajuste de sus límites, el manejo de electrodos y sensores, los procesos de escalamiento clínico y comunicación y otras acciones observables de gestión de alarmas. Las variables operativas incluyeron la frecuencia, los patrones de repetición y la duración de las alarmas, además de los tiempos de respuesta a las alarmas blancas, amarillas y rojas. En el sistema utilizado, las alarmas blancas correspondían a alertas técnicas o informativas de baja prioridad; las amarillas, a advertencias fisiológicas de prioridad media que requerían evaluación clínica; y las rojas, a alarmas críticas de alta prioridad que exigían una respuesta clínica inmediata. Las variables perceptuales comprendieron los niveles de fatiga por alarmas. En conjunto, estas variables representaron los componentes demográficos, conductuales, perceptuales y operativos pertinentes para evaluar el desempeño en la gestión de alarmas y la fatiga por alarmas.

### Instrumentos de Recolección de Datos

Formulario de identificación del personal de enfermería: Los investigadores elaboraron este formulario de 14 ítems, a partir de la literatura, para recopilar información sobre las características demográficas y profesionales de los participantes^(1,14,15)^.

Paquete CEASE: Desarrollado por Lewis y Oster^(15)^, el paquete CEASE es un conjunto de medidas de monitoreo específicas para cada paciente, dirigidas por enfermería y basadas en la evidencia, diseñado para reducir la fatiga por alarmas. Comunicación (C) se refiere al intercambio de información sobre los parámetros y problemas relacionados con las alarmas; Electrodos (E), al reemplazo diario de los electrodos; Adecuación (A), a la evaluación de la pertinencia del monitoreo; Configuración (S), a los ajustes de los parámetros de alarma específicos para cada paciente; y Educación (E), a la capacitación continua. En este estudio, el paquete CEASE orientó la observación y evaluación de las conductas de gestión de alarmas en los grupos de intervención y control.

Formulario de tipo y gestión de alarmas: Los investigadores elaboraron este formulario a partir de una revisión de la literatura^(5,16)^. Incluía apartados sobre el tipo y la definición de la alarma, los parámetros, la respuesta, el número de repeticiones y el tiempo de respuesta.

Herramienta de control de alarmas del monitor «Remind Me»: Los investigadores elaboraron esta herramienta con base en las recomendaciones de la Association for the Advancement of Medical Instrumentation (AAMI), The Joint Commission (TJC) y la American Association of Critical-Care Nurses (AACN)^(6,9,16)^. La herramienta consta de cuatro apartados: comprobación del monitor, exactitud de los ajustes de alarma, identificación de los tipos de alarma y documentación. Se diseñó para complementar el componente Educación del paquete CEASE y mantener visibles los pasos clave de la gestión de alarmas durante la atención habitual.

Escala de Fatiga por Alarmas: Ashrafi et al.^(17)^ desarrollaron esta escala de 23 ítems, con respuestas en una escala Likert de cinco puntos. La adaptación turca contiene 21 ítems y proporciona puntuaciones de 0 a 84; las puntuaciones más bajas indican una mayor fatiga por alarmas^(14)^.

### Recolección de Datos

La información basal se obtuvo mediante entrevistas presenciales y observación directa. Todos los participantes completaron el Formulario de identificación del personal de enfermería y la Escala de Fatiga por Alarmas. En cada unidad, dos investigadores observaron de forma continua los monitores de cabecera durante 24 horas a través del sistema central de monitoreo y utilizaron el paquete CEASE junto con el Formulario de tipo y gestión de alarmas para realizar un análisis de causa raíz. Las observaciones abarcaron la comunicación, el cuidado de los electrodos, la adecuación del monitoreo, la configuración de las alarmas y las necesidades educativas. Para reducir el sesgo relacionado con la observación, se informó al personal de enfermería que los datos se registrarían de forma anónima a nivel de unidad y no se utilizarían para evaluar el desempeño individual. Por tanto, CEASE se utilizó en ambos grupos como marco común de observación y evaluación, mientras que «Remind Me» se introdujo únicamente en las unidades de intervención. Después del periodo de intervención de 15 días, la Escala de Fatiga por Alarmas se administró nuevamente en ambos grupos.

### Implementación en el Grupo de Intervención

Tras la observación inicial, el equipo de investigación presentó «Remind Me» al personal de enfermería de la unidad de intervención. En breves sesiones de orientación se explicó cómo utilizar la herramienta y por qué era importante cada uno de sus elementos. Se colocó una copia laminada en PVC debajo de cada monitor para que permaneciera visible durante la atención habitual. El personal utilizó la herramienta durante 15 días. Durante este periodo, los responsables de la unidad ofrecieron recordatorios ocasionales, mientras que los investigadores no intervinieron de otra forma en la práctica clínica. Al finalizar la intervención, se realizó una segunda observación de 24 horas con el paquete CEASE y el Formulario de tipo y gestión de alarmas.

### Implementación en el Grupo de Control

El personal de enfermería de las unidades de control continuó con sus prácticas habituales de monitoreo y gestión de alarmas. Tras el mismo intervalo de 15 días, se realizó una segunda observación de 24 horas en estas unidades mediante el paquete CEASE y el Formulario de tipo y gestión de alarmas.

### Análisis de Datos

Los análisis se realizaron con IBM SPSS Statistics 22.0 (IBM Corp., Armonk, NY, EE. UU.). Las variables continuas se resumieron mediante medias y desviaciones estándar, y las categóricas mediante frecuencias y porcentajes. La normalidad se evaluó con la prueba de Shapiro–Wilk. Según la distribución de los datos, las comparaciones intragrupo se realizaron con la prueba t para muestras emparejadas o la prueba de rangos con signo de Wilcoxon, y las comparaciones entre grupos con la prueba t para muestras independientes o la prueba U de Mann–Whitney. Las variables categóricas se analizaron con las pruebas de chi-cuadrado o de McNemar. Se ajustó un modelo de regresión logística multivariable para identificar los factores asociados con la mejora de la gestión de alarmas. Se estableció un nivel de significación de p < 0,05.

### Aspectos Éticos

El estudio se llevó a cabo de conformidad con las directrices éticas nacionales e internacionales y fue aprobado por el Comité de Ética en Investigación de la Universidad de Fenerbahçe (16 de abril de 2024; Protocolo n.º 56.2024fbu). Todos los participantes otorgaron su consentimiento informado por escrito. El estudio cumplió los principios de la Declaración de Helsinki. Una vez finalizada la recolección de datos, la herramienta «Remind Me» se puso a disposición de las unidades de control.

### Uso de Inteligencia Artificial

Las herramientas de inteligencia artificial se utilizaron únicamente para la edición lingüística y el formato del texto. Los autores asumen plena responsabilidad por el contenido y las conclusiones. No se utilizaron herramientas de inteligencia artificial para analizar ni interpretar los datos.

## RESULTADOS

No se observaron diferencias significativas entre los grupos de intervención y control en cuanto a edad (25,07 ± 2,44 frente a 26,67 ± 4,24 años), género, estado civil, tener hijos, nivel educativo, experiencia clínica total, función clínica o percepción de las alarmas de los monitores (p > 0,05). Los indicadores CEASE posintervención mostraron que el personal del grupo de intervención suspendía las alarmas antes de los procedimientos, cancelaba parámetros innecesarios y ajustaba los límites de alarma con mayor frecuencia que el grupo control (p < 0,05; Tabla 1A). Las comparaciones pretest–postest en el grupo de intervención mostraron mejoras significativas en la identificación correcta de las alarmas blancas y amarillas, el ajuste de parámetros específicos para cada paciente, el número de alarmas repetidas y los tiempos de respuesta (p < 0,05; Tabla 1B); las reducciones de los tiempos de respuesta se muestran en la Figura 1. En el grupo control no se observaron cambios pretest–postest estadísticamente significativos (p > 0,05). Como durante el periodo de observación no se registraron alarmas rojas en el grupo control, sus tiempos de respuesta se presentan únicamente de forma descriptiva para el grupo de intervención y se indican como no aplicables (N/A) para el grupo control en la Tabla 1B.

**Tabla 1:** Indicadores conductuales de CEASE posintervención, resultados de respuesta a las alarmas antes y después de la intervención y predictores de mejora del desempeño en la gestión de alarmas en el personal de enfermería de cuidados intensivos (N = 115) – Estambul, Turquía, 2024.

A. Comparación entre grupos de los indicadores conductuales de CEASE posintervención*			
**Componente de CEASE**	**Grupo de intervención posintervención % (n = 62)**	**Grupo control posintervención % (n = 53)**	**p^†^**
Alarma suspendida antes de los procedimientos	83,3	61,2	**0,041**
Parámetros innecesarios cancelados	68,4	50,0	**0,046**
Límites de alarma ajustados según necesidad	76,3	58,9	**0,039**
**B. Desempeño de respuesta a las alarmas antes y después, según el tipo de alarma**			
**Tipo de alarma e indicador de respuesta**	**Grupo de intervención, pretest → postest**	**Grupo control, pretest → postest**	**p^‡^**
Alarma blanca – Identificación correcta	82,0% → 88,5%	69,2% → 69,2%	**0,040**
Alarma blanca – Parámetros específicos para cada paciente ajustados	78,7% → 96,7%	65,4% → 65,4%	**0,001**
Alarma blanca – Alarma resuelta	13,1% → 37,7%	(Sin datos)	N/A^§^
Alarma blanca – Alarmas repetidas (media ± DE)	18,38 ± 5,74 → 17,00 ± 5,27	19,73 ± 5,90 → 19,73 ± 5,90	**0,009**
Alarma blanca – Tiempo de respuesta, s (media ± DE)	43,0 ± 19,4 → 35,2 ± 15,1	45,7 ± 26,6 → 46,1 ± 26,6	**0,018**
Alarma amarilla – Identificación correcta	49,2% → 62,3%	67,3% → 68,7%	**0,002**
Alarma amarilla – Parámetros específicos para cada paciente ajustados	86,9% → 96,7%	76,9% → 76,9%	**0,004**
Alarma amarilla – Alarma resuelta	Sin datos → 29,5%	(Sin datos)	N/A^§^
Alarma amarilla – Alarmas repetidas (media ± DE)	15,31 ± 3,76 → 13,80 ± 3,24	18,19 ± 5,74 → 18,19 ± 5,74	**0,001**
Alarma amarilla – Tiempo de respuesta, s (media ± DE)	35,5 ± 16,5 → 30,1 ± 13,8	56,5 ± 24,6 → 56,5 ± 24,6	**0,021**
Alarma roja – Identificación correcta	16,4% → 18,0%	(Sin alarmas rojas)	N/A^§^
Alarma roja – Tiempo de respuesta, s (media ± DE)	13,0 ± 6,7 → 10,0 ± 4,3	(Sin datos)	N/A^§^
**C. Predictores multivariables de la mejora del desempeño en la gestión de alarmas**			
**Variable independiente**	**^||^β (EE)**	**^¶^OR (IC del 95%)**	p**
Intervención «Remind Me»	1,37 (0,52)	3,95 (1,38–11,32)	**0,008**
Experiencia (años)	0,06 (0,03)	1,06 (1,00–1,13)	**0,048**
Capacitación previa sobre alarmas (sí)	0,85 (0,41)	2,34 (1,05–5,24)	**0,039**
Horas de trabajo semanales	–0,04 (0,02)	0,96 (0,92–0,99)	**0,042**

*CEASE = Comunicación, Electrodos, Adecuación, Configuración, Educación;

^†^pruebas de McNemar y chi-cuadrado;

^‡^pruebas de rangos con signo de Wilcoxon y U de Mann–Whitney;

^§^no aplicable (N/A);

^||^β = coeficiente beta; EE = error estándar;

^¶^OR = odds ratio; IC = intervalo de confianza;

**modelo de regresión logística binaria.

Se estableció un nivel de significación de p < 0,05.

**Figura 1:** Tiempos de respuesta a las alarmas (segundos) según el tipo de alarma en el personal de enfermería de cuidados intensivos – Estambul, Turquía, 2024.

El análisis de regresión logística multivariable mostró que la intervención «Remind Me» se asoció con un mejor desempeño en la gestión de alarmas (OR = 3,95; IC del 95%: 1,38–11,32; p = 0,008). La experiencia profesional (OR = 1,06; IC del 95%: 1,00–1,13; p = 0,048) y la capacitación previa sobre alarmas (OR = 2,34; IC del 95%: 1,05–5,24; p = 0,039) también se asociaron positivamente con la mejora del desempeño, mientras que las horas de trabajo semanales mostraron una asociación inversa (OR = 0,96; IC del 95%: 0,92–0,99; p = 0,042) (Tabla 1C).

La Figura 2 muestra que los tiempos de respuesta disminuyeron para las alarmas blancas, amarillas y rojas en el grupo de intervención, mientras que en el grupo control el cambio fue mínimo o nulo para las alarmas blancas y amarillas. No se calculó el cambio porcentual del tiempo de respuesta a las alarmas rojas en el grupo control porque no se observaron alarmas de este tipo durante el periodo de estudio.

**Figura 2:** Cambio porcentual de los tiempos de respuesta a las alarmas entre el pretest y el postest, según el tipo de alarma, en el personal de enfermería de cuidados intensivos – Estambul, Turquía, 2024.

Las puntuaciones de la Escala de Fatiga por Alarmas aumentaron en el grupo de intervención desde el pretest (68,67 ± 7,52) hasta el postest (74,92 ± 4,22), mientras que en el grupo control no se observó un cambio estadísticamente significativo (Tabla 2). Las puntuaciones postest difirieron significativamente entre los grupos (p = 0,005). También se observaron diferencias significativas entre grupos en los siguientes componentes de CEASE: suspensión de alarmas antes de los procedimientos, comunicación de la alarma al profesional de enfermería responsable, cuidado de los electrodos y del oxímetro de pulso, cancelación de parámetros innecesarios, ajuste adecuado de los límites de alarma, modificación de la configuración según el estado clínico del paciente e identificación de necesidades de capacitación adicional (p < 0,05; Tabla 2).

**Tabla 2:** Comparación de las puntuaciones de la Escala de Fatiga por Alarmas y de los componentes de CEASE con diferencias significativas en el personal de enfermería de cuidados intensivos (N = 115) – Estambul, Turquía, 2024.

Parámetro	Intervención (%/media ± DE)	Control (%/media ± DE)	p*
**Escala de Fatiga por Alarmas**			
Puntuación pretest	68,67 ± 7,52	70,92 ± 8,69	**< 0,001**
Puntuación postest	74,92 ± 4,22	71,08 ± 8,72	**0,005**
**Componentes de CEASE con diferencias significativas†**			
Alarma suspendida antes de los procedimientos	93,4% / 74,9 ± 4,2	78,8% / 71,0 ± 6,1	**0,041**
Alarma comunicada al profesional de enfermería responsable	85,2% / 75,2 ± 3,9	63,5% / 70,5 ± 6,0	**< 0,001**
Cuidado de los electrodos y del oxímetro de pulso	85,2% / 74,8 ± 4,0	80,8% / 70,9 ± 6,1	**0,049**
Parámetros innecesarios cancelados	83,6% / 75,0 ± 3,8	76,9% / 70,1 ± 6,0	**0,046**
Límites de alarma ajustados adecuadamente	85,2% / 74,7 ± 4,3	78,8% / 71,5 ± 5,9	**0,025**
Configuración ajustada según el estado clínico	83,6% / 74,5 ± 4,1	76,9% / 70,9 ± 6,3	**0,039**
Necesidad de capacitación adicional identificada	78,7% / 75,1 ± 3,9	63,5% / 69,8 ± 6,5	**0,031**

*Prueba t para muestras emparejadas;

†CEASE = Comunicación, Electrodos, Adecuación, Configuración, Educación;

^‡^pruebas de McNemar y chi-cuadrado.

Se estableció un nivel de significación de p < 0,05.

## DISCUSIÓN

Las unidades de cuidados intensivos generan cada día un gran número de alarmas que no requieren una intervención clínica, lo que contribuye tanto a la fatiga por alarmas del personal de enfermería como al retraso en la respuesta ante emergencias clínicas reales^(6,10,11,18)^. La Alerta de Evento Centinela n.º 50 de The Joint Commission recomienda realizar un análisis de la situación y desarrollar protocolos específicos para cada unidad con el fin de mejorar la seguridad de las alarmas^(5)^. En consonancia con estas recomendaciones, la incorporación de «Remind Me» —un recordatorio conductual de bajo costo diseñado para reforzar el componente educativo del paquete CEASE— a las prácticas habituales de monitoreo se asoció con tiempos de respuesta más breves, mejores conductas de gestión de alarmas y puntuaciones más favorables en la Escala de Fatiga por Alarmas.

En el grupo de intervención, los tiempos de respuesta disminuyeron aproximadamente entre el 15% y el 23% según el tipo de alarma. Lewis y Oster^(15)^ comunicaron una reducción del 31% en la frecuencia total de alarmas tras implementar el paquete CEASE. Aunque este estudio se centró en los tiempos de respuesta y Lewis y Oster^(15)^ analizaron la frecuencia de las alarmas, ambos resultados reflejan mejoras en dimensiones diferentes pero complementarias de su gestión. El paquete CEASE se utilizó como marco de observación y evaluación en ambos grupos; sin embargo, los tiempos de respuesta mejoraron únicamente en el grupo de intervención, donde se aplicó «Remind Me». Este hallazgo indica que el recordatorio puede reforzar las prácticas habituales de gestión de alarmas.

La mejora de las conductas de gestión de alarmas indica que «Remind Me» pudo ayudar al personal de enfermería a realizar estas prácticas con mayor constancia. El efecto fue especialmente evidente en conductas directamente observables, como suspender las alarmas antes de los procedimientos, cancelar parámetros innecesarios y ajustar los límites de alarma ante cambios en el estado clínico del paciente. Estas acciones requieren no solo conocimientos técnicos sobre la configuración de los monitores, sino también atención sostenida, reevaluación oportuna y capacidad de decisión clínica. Estudios anteriores han mostrado que la respuesta del personal de enfermería a las alarmas depende de su relevancia clínica, la carga de falsas alarmas y las prácticas utilizadas para gestionarlas^(18)^. Las estrategias basadas en la evidencia pueden reducir la carga de alarmas^(19,20)^, y la capacitación puede disminuir la fatiga por alarmas y mejorar la sensibilización y las conductas del personal^(21)^. Asimismo, factores contextuales como la carga de trabajo, la cantidad de alarmas y la personalización de los monitores pueden influir en estos procesos^(1,10,13)^; además, se ha demostrado que una mayor carga de trabajo perjudica la percepción y localización de las alarmas^(22)^.

Al analizar los resultados por tipo de alarma, el efecto de «Remind Me» fue más evidente en las alarmas de prioridad baja y media. En las alarmas blancas, el aumento del ajuste de parámetros específicos para cada paciente indica que no solo se identificaron, sino que también se reevaluaron según el estado clínico. En las alarmas amarillas, las mejoras simultáneas en la identificación correcta, el ajuste de parámetros, el número de alarmas repetidas y el tiempo de respuesta indican que «Remind Me» pudo favorecer una respuesta más sistemática ante alteraciones fisiológicas de prioridad media. Estos resultados coinciden con estudios que muestran que la capacitación y las estrategias estructuradas pueden reducir la carga de alarmas y mejorar las conductas de gestión^(19,21)^. En las alarmas rojas, la disminución del tiempo de respuesta de 13,0 a 10,0 segundos es clínicamente relevante porque indica una orientación más rápida hacia las alarmas de alta prioridad. No obstante, al no disponer de observaciones comparables en el grupo control, este resultado debe interpretarse como una tendencia clínica y no como una comparación estadística. En conjunto, el patrón observado indica que «Remind Me» puede mejorar la gestión de las alarmas de prioridad baja y media, reducir la exposición a alarmas repetidas y preservar la atención clínica para las alarmas de alta prioridad.

El análisis multivariable respalda la asociación independiente entre «Remind Me» y la mejora del desempeño en la gestión de alarmas. La pertenencia al grupo de intervención se asoció con odds de mejora casi cuatro veces mayores, mientras que la experiencia profesional y la capacitación previa sobre alarmas actuaron como predictores positivos. Por el contrario, un mayor número de horas de trabajo semanales se asoció con menores odds de mejora, lo que indica que la gestión de alarmas depende no solo del conocimiento y la sensibilización individuales, sino también de las condiciones laborales y la carga de trabajo. Este resultado coincide con estudios que muestran que la capacitación puede reducir la fatiga por alarmas y mejorar la sensibilización y las conductas^(21)^, así como con investigaciones que señalan la influencia de la carga de alarmas, la personalización de los monitores y otros factores contextuales en su gestión^(1,13,22)^.

La fatiga por alarmas reduce la sensibilidad del personal de enfermería ante las señales de emergencia reales, aumenta el riesgo de omitir alarmas críticas y compromete la seguridad del paciente^(23,24)^. Las alarmas que no requieren intervención y las alarmas repetidas se asocian con distracción, desensibilización y retrasos en la respuesta^(1,4,6,13)^, y el propio personal ha señalado que la fatiga por alarmas retrasa su respuesta^(5,10)^. Dado que una puntuación más alta en la Escala de Fatiga por Alarmas representa un resultado más favorable, la mayor puntuación postest del grupo de intervención indica que «Remind Me» pudo reducir la fatiga por alarmas. En el grupo control, la adherencia superó el 75% en varios indicadores relacionados con CEASE, lo que indica que el paquete pudo apoyar las conductas básicas de gestión. No obstante, las tasas de adherencia más altas del grupo de intervención señalan que «Remind Me» pudo facilitar en mayor medida la incorporación de las recomendaciones de CEASE a la atención habitual. Así, la mejora de las puntuaciones de la escala se produjo junto con cambios conductuales observables.

En conjunto, estos resultados indican que «Remind Me» pudo actuar como una señal visual sencilla a pie de cama y facilitar la incorporación de las recomendaciones de CEASE a la práctica habitual. Los recordatorios conductuales pueden ayudar a mantener los efectos de la capacitación, especialmente cuando el impacto de una formación estructurada disminuye con el tiempo^(20,21)^. También se ha observado que las señales visuales y las intervenciones basadas en recordatorios fortalecen las conductas de gestión de alarmas basadas en la evidencia al apoyar la conciencia situacional y la capacidad para priorizarlas^(11)^. Por tanto, los recordatorios conductuales de bajo costo, como «Remind Me», pueden constituir una estrategia práctica para favorecer una gestión sostenible de las alarmas en las condiciones reales de las unidades de cuidados intensivos.

## LIMITACIONES

Como la asignación se basó en las estructuras clínicas existentes y no en la aleatorización, no puede descartarse la confusión residual. Para reducir este riesgo, se seleccionaron unidades con perfiles de pacientes y sistemas de monitoreo comparables.

El conocimiento de que estaban siendo observados también pudo modificar las conductas del personal de enfermería, pese a que se informó que los datos se analizarían de forma anónima a nivel de unidad. Además, no se registró con qué frecuencia ni con qué constancia se consultaba «Remind Me». Aunque la herramienta se mantuvo visible y su uso se reforzó durante reuniones informativas breves, futuros estudios deberían incorporar una medida objetiva de adherencia.

Las alarmas rojas fueron poco frecuentes durante la observación; por ello, sus tiempos de respuesta se resumieron de forma descriptiva y no se incluyeron en los análisis inferenciales.

## CONCLUSIÓN

La incorporación de «Remind Me» al paquete CEASE pareció facilitar la aplicación de este último en la práctica habitual de las unidades de cuidados intensivos. En la unidad de intervención, el personal comunicó las alarmas con mayor frecuencia al profesional de enfermería responsable, ajustó la configuración a cada paciente, canceló parámetros innecesarios e identificó necesidades de capacitación adicional. También respondió con mayor rapidez y obtuvo puntuaciones postest más favorables en la Escala de Fatiga por Alarmas.

Los resultados respaldaron las dos hipótesis planteadas e indican que el enfoque combinado puede mejorar los procesos de gestión de alarmas y contribuir a reducir la fatiga por alarmas. Aunque el número absoluto de alarmas no fue el resultado principal, los cambios observados en las conductas, los indicadores de repetición y los patrones de respuesta apuntan a una gestión más focalizada y eficaz.

Un recordatorio visual a pie de cama es económico y fácil de incorporar a los flujos de trabajo existentes. Cuando se utilizan junto con una capacitación estructurada, estos recordatorios pueden ayudar al personal de enfermería a mantener presentes los pasos esenciales de la gestión de alarmas, priorizarlas con mayor eficacia y reducir su carga acumulada en las unidades de cuidados intensivos.

## Data Availability

Los conjuntos de datos generados y/o analizados durante este estudio están disponibles previa solicitud razonable al autor correspondiente.
